# Assessment of Interrater Reliability and Accuracy of Cerebral Aneurysm Morphometry Using 3D Virtual Reality, 2D Digital Subtraction Angiography, and 3D Reconstruction: A Randomized Comparative Study

**DOI:** 10.3390/brainsci14100968

**Published:** 2024-09-26

**Authors:** Attill Saemann, Daniel de Wilde, Jonathan Rychen, Michel Roethlisberger, Marek Żelechowski, Balázs Faludi, Philippe Claude Cattin, Marios-Nikos Psychogios, Jehuda Soleman, Raphael Guzman

**Affiliations:** 1 Department of Neurosurgery, University Hospital of Basel, 4031 Basel, Switzerland; 2 Faculty of Medicine, University of Basel, 4056 Basel, Switzerland; 3 Department of Biomedical Engineering, University of Basel, 4123 Allschwil, Switzerland; 4 Department of Neuroradiology, University Hospital Basel, 4031 Basel, Switzerland

**Keywords:** augmented reality, cerebrovascular neurosurgery, intracranial aneurysm, measurement, morphometry, surgical planning, virtual reality

## Abstract

Background/Objectives: Detailed morphometric analysis of an aneurysm and the related vascular bifurcation are critical factors when determining rupture risk and planning treatment for unruptured intracranial aneurysms (UIAs). The standard visualization of digital subtraction angiography (DSA) and its 3D reconstruction on a 2D monitor provide precise measurements but are subject to variability based on the rater. Visualization using virtual (VR) and augmented reality platforms can overcome those limitations. It is, however, unclear whether accurate measurements of the aneurysm and adjacent arterial branches can be obtained on VR models. This study aimed to assess interrater reliability and compare measurements between 3D VR, standard 2D DSA, and 3D DSA reconstructions, evaluating the reliability and accuracy of 3D VR as a measurement tool. Methods: A pool of five neurosurgeons performed three individual analyses on each of the ten UIA cases, measuring them in completely immersed 3D VR and the standard on-screen format (2D DSA and 3D reconstruction). This resulted in three independent measurements per modality for each case. Interrater reliability of measurements and morphology characterization, comparative differences, measurement duration, and VR user experience were assessed. Results: Interrater reliability for 3D VR measurements was significantly higher than for 3D DSA measurements (3D VR mean intraclass correlation coefficient [ICC]: 0.69 ± 0.22 vs. 3D DSA mean ICC: 0.36 ± 0.37, *p* = 0.042). No significant difference was observed between 3D VR and 2D DSA (3D VR mean ICC: 0.69 ± 0.22 vs. 2D DSA mean ICC: 0.43 ± 0.31, *p* = 0.12). A linear mixed-effects model showed no effect of 3D VR and 3D DSA (95% CI = −0.26–0.28, *p* = 0.96) or 3D VR and 2D DSA (95% CI = −0.02–0.53, *p* = 0.066) on absolute measurements of the aneurysm in the anteroposterior, mediolateral, and craniocaudal dimensions. Conclusions: 3D VR technology allows for reproducible, accurate, and reliable measurements comparable to measurements performed on a 2D screen. It may also potentially improve precision for measurements of non-planar aneurysm dimensions.

## 1. Introduction

Unruptured intracranial aneurysms (UIAs), relatively common vascular abnormalities found in 2–4% of the population [1], are increasingly detected due to the widespread use of non-invasive imaging [2]. While most UIAs remain asymptomatic, with a low risk of rupture, some may eventually rupture and lead to subarachnoid hemorrhage (SAH), which is associated with substantial morbidity and mortality [3]. The management of UIAs includes three primary approaches: conservative (with follow-up imaging), endovascular, or surgical treatment [4]. Selecting the most suitable strategy is a complex, patient-specific, and multidisciplinary decision. This decision is grounded in a comprehensive assessment comparing potential interventional and rupture risks.

Rupture risk is based on aneurysm dimensions, morphological features, and clinical patient characteristics [1,5,6,7]. In clinical practice, aneurysm dimensions and morphological features are necessary to calculate validated risk assessment scores such as PHASES and UIATS [7,8,9] and morphological parameters such as size ratio, aspect ratio, and dome-to-neck ratio, objectively quantifying rupture risk [10,11,12]. These scores have limits; for instance, they tend to inadequately assess small aneurysms (<7 mm), highlighting the need for a detailed evaluation of aneurysm dynamics and morphological characteristics [13,14,15,16].

Developed through different methodologies, these morphological parameters each provide a unique perspective: The aspect ratio is based on 2D digital subtraction angiography (DSA) measurements [10,17], while the size ratio and dome-to-neck ratio are derived from 3D DSA reconstruction measurements [11,12]. Ultimately, a precise assessment of the aneurysm is invaluable for planning the treatment (i.e., clip placement or fitting of an endosaccular device).

In a typical clinical routine, these measurements and characterizations are performed on 2D screens based on the primary imaging dataset, such as digital subtraction angiography (DSA), computed tomography angiography (CTA), or magnetic resonance angiography (MRA) of the UIA by neurosurgeons or neuroradiologists [18,19,20]. Among these methods, DSA remains the gold standard for analyzing aneurysms in clinical practice, with 3D DSA reconstruction (hereafter referred to as 3D DSA) preferred over 2D source imaging due to its ability to provide more detailed and 3D visualization of the aneurysm [10,21,22,23].

Virtual reality (VR) has quickly emerged as a transformative tool in the medical field, offering substantial benefits across applications, including medical education, patient education, rehabilitation, and surgical planning [24,25,26,27,28]. One of VR’s key advantages is its ability to generate detailed 3D models of complex anatomy, which can be viewed and manipulated from multiple angles. This capability allows clinicians and students to overcome the inherent limitations of traditional 2D imaging, offering a more comprehensive understanding of anatomy and pathology. Newer VR technologies also offer intraluminal perspectives, further underscoring that VR could provide reliable measurements and characterization of UIAs compared to the 2D DSA source images and 3D reconstructions. However, despite the increasing use and availability of VR for neurosurgical planning, the validity of VR’s measurement capability has yet to be confirmed, and its reliability in measurements and morphological characterization remains an area that requires further investigation.

To address this gap, we compared the interrater reliability of aneurysm measurements and morphological characterization of UIAs using 3D VR, 3D DSA, and 2D DSA. Furthermore, we aimed to investigate the comparative difference in size measurements and the duration of the measurements.

## 2. Materials and Methods

### 2.1. Study Design and Participants

**Figure 1:**
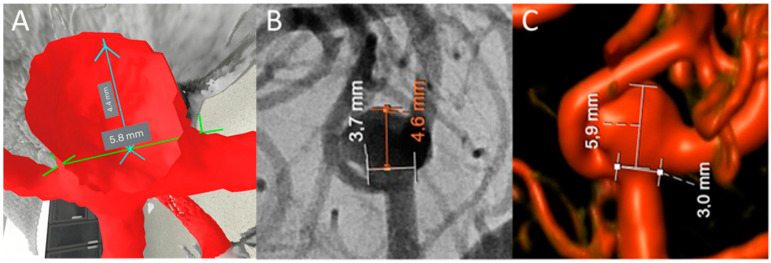
Comparison of 2D DSA, 3D DSA, and 3D VR measurements. (**A**) Measurement of neck diameter and maximum perpendicular aneurysm height using 3D VR. (**B**) Measurement of neck diameter and maximum perpendicular height using 2D DSA. (**C**) Measurement of neck diameter and maximum perpendicular aneurysm height using 3D DSA reconstructions.

Patients who underwent open surgical or endovascular treatment for UIAs at “our hospital” from 2021 to 2022 were retrospectively screened for inclusion. All of these had complete imaging and were judged to have been treated by an interdisciplinary neurovascular board consisting of neurosurgeons and interventional neuroradiologists. Ten cases were randomly selected and assigned unique identifiers. The latest 2D DSA (Siemens Healthineers, Erlangen, Germany) source images were used as baseline images for 2D DSA measurements. The vessel-injected flat-detector CTA (FD-CTA) from the same imaging session was used as a source image to reconstruct the 3D DSA and 3D VR models. Raters were randomly assigned to the UIA cases using an online randomization tool (randomlists.com, FL, USA). Each rater followed a measuring protocol (see Supplemental Digital Content S1 and S2) in the 3D VR and 2D modalities (Figure 1). The raters included five microsurgically trained neurosurgeons from the Department of Neurosurgery at the University Hospital of Basel, with various degrees of experience in vascular neurosurgery (one chief resident, three attending vascular neurosurgeons, and one chairman of vascular neurosurgery).

### 2.2. VR Software

Each FD-CTA DICOM dataset was converted into a 3D VR model within the VR software SpectoVR (Version 5.0.0, Specto Medical AG, Basel, Switzerland). Initially, the DICOM dataset was imported into the software, followed by Hounsfield unit-based segmentation to visualize the blood vessels and the skull. Subsequently, an investigator manually removed any unnecessary noise or vessels to refine the model for accurate visualization. The geometrical and anatomical precision of SpectoVR has been established through previous validation studies [29,30]. The software was deployed on a Windows PC (Razer Blade 17 2022, Irvine, CA, USA, Intel CPU i7-12800H, 16GB DDR5 RAM, NVidia GeForce RTX 3080 Ti GPU, Santa Clara, CA, USA). Each model was subsequently saved and stored as a custom Specto file type on a password-encrypted external hard drive.

### 2.3. Measurements

The aneurysm dimensions measured in millimeters (mm) included the anteroposterior, mediolateral, craniocaudal, dome, neck (only if the presence of a neck was established), parent vessel diameter, maximum perpendicular height, and maximum aneurysm height. In addition to the measurements, raters were asked to assess aneurysm features, including morphological classification, the presence of a neck, spatial aneurysm orientation, and parent vessel (see Supplemental Digital Content S1). Raters first performed measurements in 3D VR, followed by measurements in 2D DSA and 3D DSA on-screen, with substantial temporal intervals between each modality.

**Figure 2:**
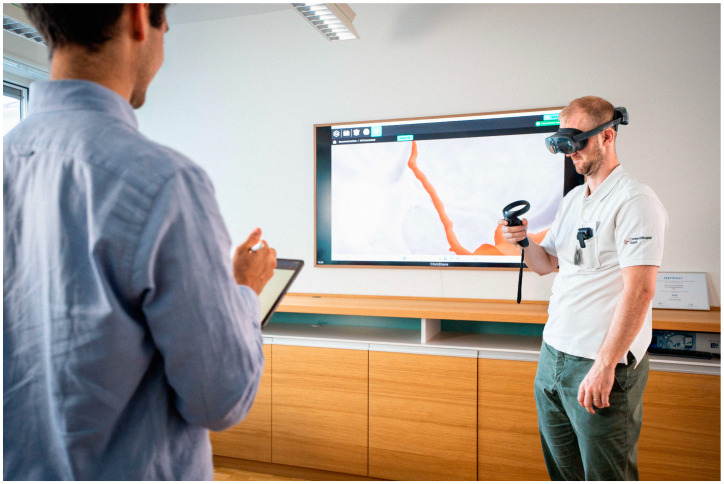
Investigator guiding rater through 3D VR measurements.

**Figure 3:**
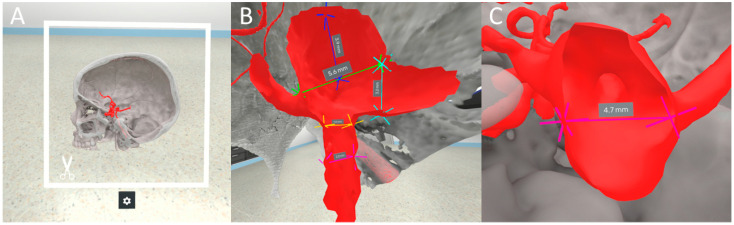
Various VR images from the study. (**A**) Interactive cutting plane feature for precise 3D slicing of anatomical models. (**B**) Measurement of neck diameter, maximum perpendicular height, and parent artery diameters in a 3D VR environment. (**C**) Intraluminal perspective for dome diameter measurement.

Before the 3D VR measurements, each rater completed a questionnaire (see Supplemental Digital Content S2) containing questions about their neurosurgical experience and prior VR exposure. Raters were additionally granted the opportunity to review an overview of the aneurysm dimensions and morphological features planned to be assessed and pose any questions regarding definitions assuring standardized measurement. Subsequently, they entered a fully immersive 3D VR space using an HP Reverb G2 headset and HP motion controllers (HP Inc., Palo Alto, CA, USA). The investigator asked all raters to measure each aneurysm dimension and assess each morphological feature individually (Figure 2). Measurements in 3D VR were performed using two custom-definable points in the 3D space (Video S3, Figure 3).

After all 3D VR measurements were completed, a questionnaire was administered to examine each rater’s 3D VR measurement experience (see Supplemental Digital Content S2). Following the 3D VR measurements, all 2D and 3D DSA measurements were performed in the Sectra PACS image viewing software (Sectra AB, Linkoping, Sweden), following the same methodology outlined for the 3D VR measurements. Across all three modalities, measurements were performed with an accuracy of 0.1 mm.

### 2.4. Outcome Variables

The study’s primary outcome was the interrater reliability in size measurement and description of aneurysm features (morphological classification, presence of neck, orientation, and parent vessel) between the three modalities (3D VR, 3D DSA, and 2D DSA).

The secondary outcomes included the comparative difference in size measurements in millimeters of the patient-specific UIA conducted in the modalities, the measurements’ duration, and the VR’s usability.

### 2.5. Statistical Analysis

All generated data were recorded in case report forms (Supplemental Digital Content S1 and S2). The data were subsequently imported as a CSV file into RStudio (Version 1.4.1106, Posit, PBC, Boston, MA, USA) for statistical analysis.

Comparative statistics were determined using the Wilcoxon signed-rank test, ANOVA, paired *t*-test, z-test, and linear mixed-effects model. Interrater reliability was assessed using the intraclass correlation coefficient (ICC) and Fleiss’ ϰ. The threshold for statistical significance was set at a *p* value < 0.05.

## 3. Results

### 3.1. Patient Population and Rater Cohort

The study included ten patient-based cases with UIAs mostly arising from the anterior circulation, particularly the middle cerebral artery (50%, *n* = 5) and anterior communicating artery (30%, *n* = 3) (Table 1). Every case was assessed by three different raters out of a pool of five neurosurgeons. Consequently, a total of 90 measurements were acquired, 30 of which were performed using 3D VR, 30 using 3D DSA, and 30 using 2D DSA.

### 3.2. Primary Outcome

#### 3.2.1. Interrater Reliability of Aneurysm Measurements Comparing 3D VR with 3D DSA and 2D DSA

**Figure 4:**
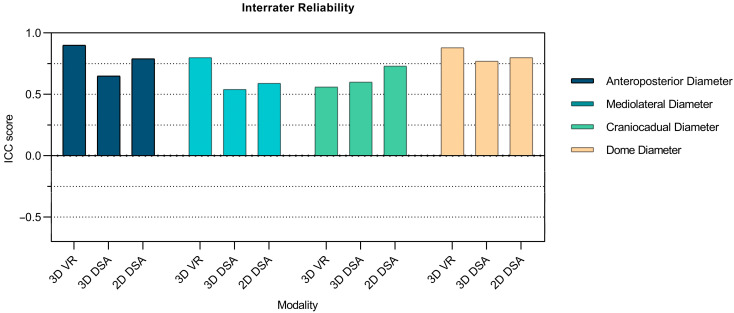
Bar charts showing ICC scores for 2D DSA, 3D DSA, and 3D VR measurements of various aneurysm dimensions.

**Figure 5:**
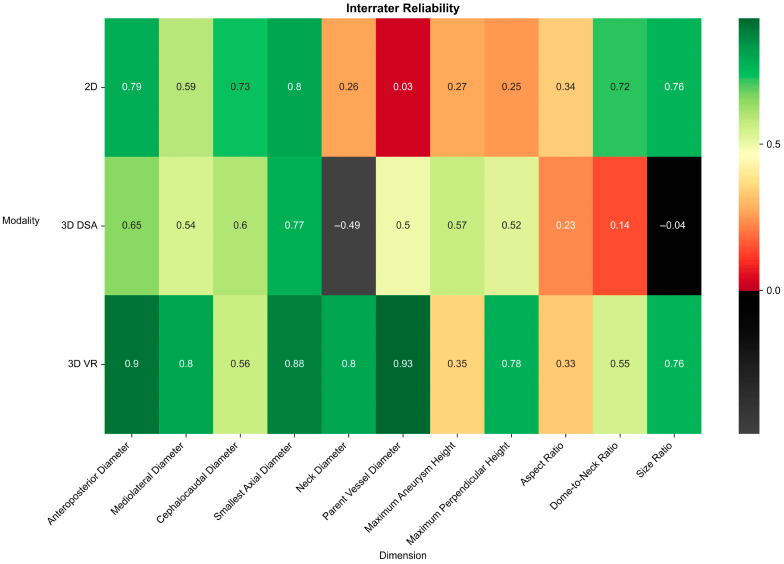
Heat map of ICC scores for 2D DSA, 3D DSA, and 3D VR measurements of various aneurysm dimensions. ICC values indicate reliability: below 0.5 = poor, 0.5–0.75 = moderate, 0.75–0.9 = good, and above 0.9 = excellent.

Measurements of the anteroposterior diameter, mediolateral diameter, dome diameter, neck diameter, parent vessel diameter, and maximum perpendicular height all showed higher interrater reliability in 3D VR than in 3D DSA and 2D DSA. Craniocaudal diameter was the only dimension to demonstrate lower interrater reliability in 3D VR than both 3D DSA and 2D DSA. Maximum aneurysm height showed lower interrater reliability in 3D VR than 3D DSA alone (Table 2, Figure 4 and Figure 5).

Overall, 3D VR exhibited higher interrater reliability than both 3D DSA and 2D DSA in aneurysm dimension measurements. The comparison between 3D VR and 3D DSA was significantly different (3D VR mean ICC: 0.69 ± 0.22 vs. 3D DSA mean ICC: 0.36 ± 0.37, *p* = 0.042). However, this difference did not reach significance when 3D VR was compared to 2D DSA (3D VR mean ICC: 0.69 ± 0.22 vs. 2D DSA mean ICC: 0.43 ± 0.31, *p* = 0.12).

#### 3.2.2. Interrater Reliability of Morphological Aneurysm Features Comparing 3D VR with 3D DSA and 2D DSA

Morphological classification displayed non-significantly higher interrater reliability in 3D VR (3D VR [Fleiss’ ϰ (95% CI)]: 0.35 (0.13–0.57)) than 3D DSA (3D DSA [ϰ (95% CI)]: 0.16 (−0.06–0.37), *p* = 0.23) and significantly higher interrater reliability than 2D DSA (2D DSA [ϰ (95% CI)]: −0.045 (−0.29–0.2), *p* = 0.019). 3D VR (3D VR [ϰ (95% CI)]: 0.26 (−0.1–0.62) exhibited non-significant higher interrater reliability when determining the presence of an aneurysm neck than 3D DSA (3D DSA [ϰ (95% CI)]: −0.05 (−0.41–0.31), *p* = 0.23) and 2D DSA (2D DSA [ϰ (95% CI)]: −0.071 (−0.45–0.31), *p* = 0.22). Regarding aneurysm orientation, 3D VR (3D VR [ϰ (95% CI)]: 0.3 (0.17–0.43)) exhibited significantly lower interrater reliability than 2D DSA (2D DSA [ϰ (95% CI)]: 0.51 (0.38–0.63), *p* = 0.02) and non-significantly higher interrater reliability than 3D DSA (3D DSA [ϰ (95% CI)]: 0.17 (0.027–0.31), *p* = 0.18). Accurate identification of the parent vessel was achieved in 90% (*n* = 27) of cases using 3D VR assessment, in 70% (*n* = 21) using 3D DSA assessment, and in 93% (*n* = 28) using 2D DSA assessment.

### 3.3. Secondary Outcomes

#### 3.3.1. Comparative Differences between the 3D VR Measurements and the 3D and 2D DSA Measurements

**Figure 6:**
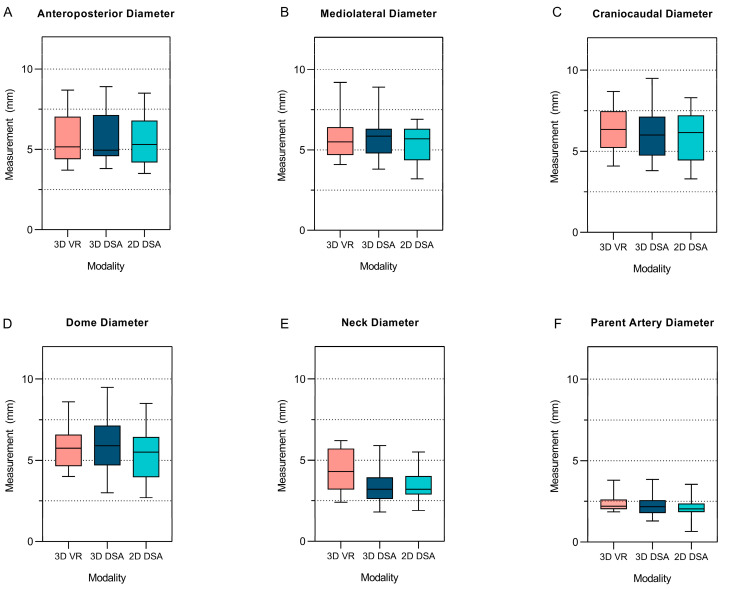
Box plots showing mean and range using 2D DSA, 3D DSA, and 3D VR for measurements of various aneurysm dimensions. (**A**) Anteroposterior diameter. (**B**) Mediolateral diameter. (**C**) Craniocaudal diameter. (**D**) Dome diameter. (**E**) Neck diameter. (**F**) Parent artery diameter.

All dimensions and morphological parameters except for dome diameter, maximum height, maximum perpendicular height, and dome-to-neck ratio showed non-significant differences between 3D VR and 3D DSA (Table 3, Figure 6). Compared to 2D DSA, all dimensions and morphological parameters, except for neck diameter, parent vessel diameter, maximum height, and perpendicular height, showed non-significant differences in 3D VR (Table 3, Figure 6).

A linear mixed-effects model comparing the effect of 3D VR and 3D DSA for anteroposterior, mediolateral, and craniocaudal diameter measurements found no significant difference (95% CI = −0.26–0.28, *p* = 0.96). Also, the model indicated no significant difference in diameter measurements between 3D VR and 2D DSA (95% CI = −0.02–0.53, *p* = 0.066).

#### 3.3.2. Measurement Duration

When comparing 3D VR and 2D DSA, the 3D VR measurements were found to have a non-significantly higher mean duration (3D VR mean duration: 8.2 ± 15 min vs. 2D DSA mean duration: 7.1 ± 11 min, *p* = 0.15). The mean measurement duration was significantly higher for 3D VR measurements than for 3D DSA measurements (3D VR mean duration: 8.2 ± 15 min vs. 3D DSA mean duration: 4.7 ± 5 min, *p* = 0.000006).

**Figure 7:**
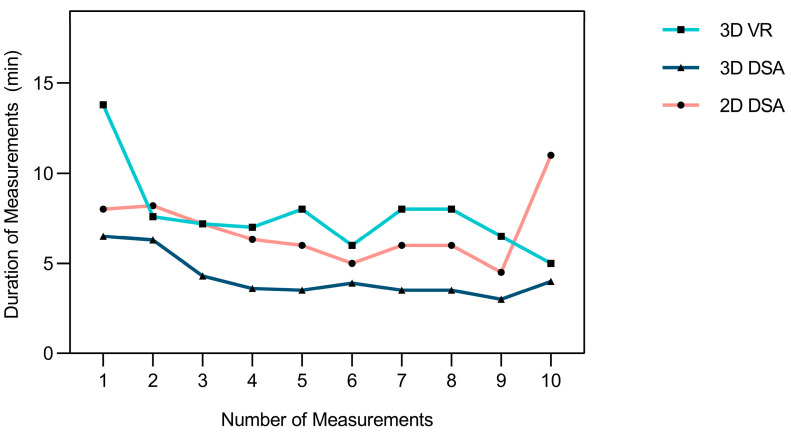
Line chart showing mean duration of measurement in relation to number of measurements performed.

The mean slope for measurement duration was higher for 3D VR than for 3D DSA and 2D DSA (3D VR: −1.14 ± 9 vs. 3D DSA: −0.66 ± 1.6 vs. 2D DSA: −0.41 ± 1.7), indicating a positive learning curve with increased usage (Figure 7). However, no statistically significant differences were noted between 3D VR and 3D DSA (*p* = 0.76) or between 3D VR and 2D DSA (*p* = 0.65).

#### 3.3.3. Subjective Rater Experience

All raters described the 3D VR models as more intuitive to assess than the standard on-screen measurements. Furthermore, each rater expressed a desire to utilize 3D VR technology more frequently. Each rater observed phases of flow and immersion during their 3D VR measurements, with no motion sickness reported (Table 4).

## 4. Discussion

Our results have shown that 3D VR measurements and the morphological characterization of aneurysms tend to exhibit higher interrater reliability than 2D DSA source images and their derived 3D reconstructions. These findings promote the potential utility of 3D VR technology as a modern tool for reproducible, precise, and reliable measurement of UIAs. Furthermore, no significant differences were found in the basic anteroposterior, mediolateral, and craniocaudal diameter measurements when comparing 3D VR to 3D DSA and 2D DSA, verifying its precision and applicability in clinical practice. Morphological parameters, including size and aspect ratio, also showed no significant difference between 3D VR, 3D DSA, and 2D DSA. To the best of our knowledge, this study is the first to compare and validate aneurysm measurements performed in a fully immersed 3D VR environment with measurements conducted on traditional 2D DSA images and 3D reconstructions. 3D DSA is widely regarded as the gold standard for assessing intracranial aneurysms, offering high-resolution 3D reconstructions that allow for precise assessment of aneurysm morphology and dimensions [31,32]. However, a comparison to 3D VR suggests that 3D VR may provide more reliable measurements than the current gold standard.

The interrater reliability of 3D VR was significantly higher than that of 3D DSA and comparable to that of 2D DSA. 3D VR measurements demonstrated higher interrater reliability in seven of eight aneurysm dimensions assessed compared to 2D DSA measurements. This may be attributed to the improved 3D visualization, which eliminates spatial superimposition, making measurements more accessible and reproducible with less investigator dependency. The comparison between 3D DSA and 2D DSA revealed no significant difference in interrater reliability. This pattern aligns with the findings of Timmins et al. [32], who also observed comparable interrater reliability when comparing 2D MRAs and their 3D reconstruction on-screen. The non-significant difference in anteroposterior, mediolateral, and craniocaudal diameters (i.e., planar dimensions of an aneurysm) between 3D VR models, 2D source images, and 3D reconstructions shows that 3D VR technology can perform planar aneurysm measurements with the same precision as on-screen measurements. This finding aligns with previous studies investigating the comparability of measurements between reconstructed 3D imaging and source imagery [33].

Regarding interrater reliability for morphological aneurysm features, we found that morphological classification exhibited non-significantly higher interrater reliability in 3D VR than in 3D DSA and significantly higher interrater reliability than in 2D DSA. The accuracy of identifying parent arteries was comparable between 3D VR and 2D DSA, whereas 3D DSA was substantially less accurate. This inaccuracy may stem from the fact that only the region of interest (ROI) surrounding the aneurysm is reconstructed in 3D, which makes it challenging to understand the full anatomy and orientation in the 3D space. Additionally, comparably low interrater reliability was observed when determining the presence of a neck in all three modalities. However, it must be noted that the ICC for 3D VR was substantially higher than that observed for 3D DSA and 2D DSA. This previously reported discrepancy in determining neck presence [34] may be explained by certain raters’ tendency to infer the presence of a neck even when it may not be clearly visible. This discrepancy seemed to emerge in cases where branching vessels arise from the neck.

Interestingly, some significant differences were observed in the arguably more complex (non-planar) measurements of dome diameter, neck diameter, parent vessel diameter, maximum height, and maximum perpendicular height between 3D VR and 3D DSA and between 3D VR and 2D DSA. Upon initial assessment, this finding might lead to the conclusion that 3D VR has a diminished capability to perform such complex measurements. However, we hypothesize that the inability of 2D visualized DSAs to clearly depict aneurysm neck and parent vessel delineation substantially decreased the rater’s precision in performing neck and parent vessel diameter measurements, as observed by Hochmuth et al. [21]. In contrast, the ability of 3D VR to view and measure aneurysms independently from any angle or plane and provide intraluminal visualization suggests that 3D VR can provide more precise and realistic measurements of the diameter of the neck and parent vessel. These findings indicate that 3D VR is a tool that offers comparable planar measurements while improving precision for nonplanar aneurysm dimensions with higher interrater reliability. Thus, its measurements might be closer to the ground truth.

Higher measurement durations were found for 3D VR and 2D DSA than for 3D DSA. However, it must be noted that 3D DSA measurements are performed routinely in clinical practice; therefore, raters had more experience measuring the specific dimensions in this particular sequence than for the other two modalities. More interestingly, a higher mean rate of change in measurement duration per additional measurement performed was observed in 3D VR than for both 3D DSA and 2D DSA, suggesting a positive learning curve. The observed trend of decreased duration in VR measurements aligns with the findings of the study conducted by Greuter et al. [34] that assessed the time to detect aneurysms in 3D VR. Therefore, it can be inferred that with increased training and experience, VR can likely achieve measurement durations comparable to those of 3D DSA and 2D DSA.

Raters unanimously described the 3D VR system as more intuitive than the 3D DSA and 2D DSA on-screen environments. Additionally, VR induced states of psychological flow and immersion. Notably, the raters in this study did not report any motion sickness components, possibly due to the advanced functionality of SpectoVR, which has been previously validated [29,30].

Although still emerging in cerebrovascular surgery, the ability of VR to generate accurate and interactive 3D models has proven to be a powerful tool to enhance spatial awareness and surgical precision. Studies have shown that VR significantly improves the detection of arterial anatomy in UIAs, leading to better anatomical understanding, optimized head positioning, and a more precise selection of surgical approaches [35]. Additionally, VR has been shown to reduce operating times for aneurysm clipping through more effective preoperative planning [36]. These examples are just a few of many studies highlighting the value of VR in aneurysm surgery, where it plays a crucial role in the surgical planning, execution, and teaching of complex anatomy [37,38,39,40,41].

However, as VR is increasingly implemented in clinical practice, verifying its validity compared to the current gold standard is fundamental. While 3D VR has demonstrated precise measurement values with higher interrater reliability in our study, there is currently a lack of data on its impact on clinical decision-making and patient outcomes. Since decision-making about treating UIAs remains strongly dependent on size and morphological characteristics, the precision and objective understanding of morphology gain importance, especially in small aneurysms, for instance [15]. Additionally, with the established practice of interdisciplinary decision-making for the treatment strategy of UIAs, increased interrater reliability supports objective discussion. Dedicated studies evaluating the particular impact of 3D VR in those situations are warranted to further validate the utility and applicability of 3D VR measurement and characterization in clinical practice.

### Limitations

Several limitations should be considered when interpreting this study’s findings. First, considerable variation in the number of aneurysms measured by each rater (three to ten) may have influenced correlation and reliability results. Also, no UIAs that were managed conservatively were included. Furthermore, this study had a relatively small sample size of only ten aneurysm cases, which likely does not encompass the breadth of different locations, sizes, and features of aneurysms, thus providing low power for generalizing the findings. It is essential to note that all raters were neurosurgeons rather than neuroradiologists, who more commonly perform aneurysm size measurements. Additionally, the cost of a VR system has to be considered, as it potentially limits the availability of VR in routine neurosurgical practice globally.

## 5. Conclusions

3D VR technology offers measurements comparable to those of 2D DSA and its 3D reconstruction while likely increasing interrater reliability. It can potentially improve the precision of measurements for non-planar aneurysm dimensions such as parent vessel, neck diameter, and height. More data regarding its utility in judging morphological changes in aneurysms are required.

## Figures and Tables

**Table 1:** Overview of unruptured intracranial aneurysm cases.

Patient ID	Parent Vessel	Side
1	MCA	Left
2	ACOM	Left
3	MCA	Right
4	MCA	Right
5	ACOM	Right
6	PCOM	Right
7	PCOM	Right
8	MCA	Right
9	ACOM	Left
10	MCA	Right

**Table 2:** Interrater reliability of aneurysm dimensions and morphological parameters.

	3D VR (*n* = 30)		3D DSA (*n* = 30)		2D DSA (*n* = 30)	
Aneurysm Dimension	ICC (95% CI)	*p* Value	ICC (95% CI)	*p* Value	ICC (95% CI)	*p* Value
Anteroposterior Diameter	0.9 (0.74–0.97)	0.000000003	0.65 (0.30–0.88)	0.00025	0.79 (0.51–0.94)	0.000007
Mediolateral Diameter	0.8 (0.55–0.94)	0.0000014	0.54 (0.16–0.84)	0.0026	0.59 (0.22–0.86)	0.00097
Cephalocaudal Diameter	0.56 (0.19–0.85)	0.0016	0.6 (0.24–0.87)	0.00075	0.73 (0.43–0.92)	0.00002
Smallest Axial Diameter	0.88 (0.71–0.97)	0.00000001	0.77 (0.49–0.93)	0.000005	0.8 (0.55–0.94)	0.0000013
Neck Diameter	0.8 (0.50–0.95)	0.000016	−0.49 (−0.50–−0.08)	0.98	0.26 (−0.26–0.91)	0.18
Parent Vessel Diameter	0.93 (0.82–0.98)	0.00000000004	0.5 (0.12–0.82)	0.0047	0.03 (−0.26–0.50)	0.41
Maximum Aneurysm Height	0.35 (−0.03–0.74)	0.037	0.57 (0.20–0.85)	0.0014	0.27 (−0.09–0.69)	0.079
Maximum Perpendicular Height	0.78 (0.50–0.93)	0.000004	0.52 (0.14–0.83)	0.0033	0.25 (−0.11–0.68)	0.099
**Morphological** **Parameter**						
Aspect Ratio	0.33 (−0.09–0.77)	0.066	0.23 (−0.33–0.96)	0.23	0.34 (−0.21–0.92)	0.13
Dome-to-Neck Ratio	0.55 (0.13–0.87)	0.0051	0.14 (−0.36–0.95)	0.30	0.72 (0.17–0.98)	0.0067
Size Ratio	0.76 (0.47–0.93)	0.0000086	−0.04 (−0.3–0.43)	0.55	−0.04 (0.3–0.43)	0.55

VR = virtual reality, DSA = digital subtraction angiography, ICC = intraclass correlation coefficient, CI = confidence interval.

**Table 3:** Aneurysm dimension measurement difference comparison by modality.

	3D VR (*n* = 30)	3D DSA (*n* = 30)	2D DSA (*n* = 30)	3D VR vs. 3D DSA	3D VR vs. 2D DSA	3D DSA vs. 2D DSA
Aneurysm Dimension	Mean ± Range (mm)	Mean ± Range (mm)	Mean ± Range (mm)	*p* Value	*p* Value	*p* Value
Anteroposterior Diameter	5.7 ± 5	5.8 ± 5.1	5.7 ± 5	0.84	0.84	0.68
Mediolateral Diameter	5.7 ± 5.1	5.8 ± 5.1	5.4 ± 3.7	0.62	0.69	0.46
Craniocaudal Diameter	6.3 ± 4.6	6.1 ± 5.7	5.8 ± 5	0.42	0.23	0.63
Dome Diameter	5.8 ± 4.6	6.1 ± 6.5	5.3 ± 5.8	0.004	0.14	0.85
Neck Diameter	4.4 ± 3.8	3.3 ± 4.1	3.4 ± 3.6	0.68	0.005	0.10
Parent Vessel Diameter	2.3 ± 1.8	2.3 ± 2.55	1.9 ± 2.8	0.48	0.017	0.047
Maximum Height	6.5 ± 8.5	5.8 ± 4.4	5.7 ± 3.9	0.016	0.009	0.80
Maximum Perpendicular Height	6.3 ± 4.3	5.3 ± 4.5	5.3 ± 5.3	0.016	0.004	0.95
**Morphological** **Parameter**						
Aspect Ratio	1.6 ± 2.1	1.6 ± 1.8	1.7 ± 3.3	0.63	0.38	0.93
Dome-to-Neck Ratio	1.4 ± 2.4	1.9 ± 3.2	1.7 ± 2.6	0.0044	0.14	0.60
Size Ratio	3 ± 5.2	2.5 ± 2.4	3.3 ± 10.3	0.057	0.75	0.088

VR = virtual reality, DSA = digital subtraction angiography.

**Table 4:** Subjective virtual reality experience.

	Rater Assessment
Which modality do you find easier to detect and describe aneurysms?	5/5 (100%) virtual reality model
	Overall agreement (1 = strongly agree, 2 = agree, 3 = neutral, 4 = disagree, 5 = strongly disagree)
	Mean (SD)
Everyday thoughts and concerns faded out during the measurement.	2.1 (0.75)
I experienced fatigue, eyestrain, difficulty focusing, headache, blurred vision, dizziness (eyes closed), or vertigo.	4.5 (0.54)

## Data Availability

The data supporting the findings of this study are included within the article. Any additional data or materials can be made available to the corresponding author upon reasonable request.

## Supplementary Materials

The following supporting information can be downloaded at: https://www.mdpi.com/article/10.3390/brainsci14100968/s1, Supplemental Digital Content S1: Virtual reality experience survey, Supplemental Digital Content S2: Measurement case report form, Supplemental Digital Content S3: Video demonstrating the virtual reality measurement process.
