# Supplementary material for: Assessment of Interrater Reliability and Accuracy of Cerebral Aneurysm Morphometry Using 3D Virtual Reality, 2D Digital Subtraction Angiography, and 3D Reconstruction: A Randomized Comparative Study
Source: Brain Sci. 2024 Sep 26;14(10):968. doi: 10.3390/brainsci14100968 (PMC11506597; doi:10.3390/brainsci14100968)
Supplement: Supplementary file 1 [file brainsci-14-00968-s001.zip › Supplemental Digital Content S1.pdf]

Supplemental Digital Content 2, Methods. Virtual reality experience survey

Correlating size measurements of cerebral aneurysms based on Virtual Reality "Specto Medical" versus measurements based on standard 2D CTA/DSA – VR Experience Case Report Form v1.0

Investigator_ID: ☐☐

Date: ☐☐/☐☐/☐☐☐☐
(DD/MM/YYYY)

**1. Virtual Reality Experience** *(1 and 2 are to be asked in advance to the measurements)*		
1.1	What is your level of experience with virtual reality?	☐ None<br>☐ Novice (used it before but have little experience)<br>☐ Occasional (> once a month)<br>☐ Regular User (1-3 times per week)<br>☐ Frequent (> 3 times per week)<br>☐ Daily
1.2	How often have you used virtual reality to view aneurysms pre-operatively?	☐ Never<br>☐ <5 occasions<br>☐ 5-15 occasions<br>☐ 15-30 occasions<br>☐ >30 occasions
**2. Neurosurgical Experience**		
2.1	How many years of experience do you have in neurosurgery?	☐ <3 years<br>☐ 3-6 years<br>☐ 7-12 years<br>☐ 13-18 years<br>☐ > 18 years
2.2	How many aneurysm cases have you treated in your career?	☐ 0-25 cases<br>☐ 25-75 cases<br>☐ 75-200 cases<br>☐ 200-400 cases<br>☐ > 400 cases

**3. Flow & Immersion**[(1)]		
	Statement	*1 = Strongly Agree, 2 = Agree, 3 = Neutral, 4 = Disagree, 5 = Strongly Disagree*
3.1	Everyday thoughts and concerns faded out during the measurement	1 2 3 4 5
3.2	I was more focused on the activity rather on any external distraction	1 2 3 4 5
3.3	All of my senses were totally concentrated on the activity	1 2 3 4 5
**4. Perceived Usability**[(2)]		
4.1	I think that I would like to use this VR navigation technique frequently	1 2 3 4 5
4.2	I thought the VR navigation technique was easy to use	1 2 3 4 5

4.3	I would imagine that most people would learn to use this VR navigation technique very quickly	1 2 3 4 5
4.4	I felt very confident using the VR navigation technique	1 2 3 4 5
4.5	I believe the aneurysm sizes in the VR system reflect those in the common imaging modalities (MRI and CT)	1 2 3 4 5
4.6	I feel that VR may be helpful to better understand the aneurysm and its surrounding pre-operatively	1 2 3 4 5
4.7	Which modality do you find easier to detect and describe aneurysms?	☐VR model<br>☐2D images
**5. Experience Consequence[3]**		
5.1	I experienced fatigue	1 2 3 4 5
5.2	I experienced eyestrain	1 2 3 4 5
5.3	I experienced difficulty focusing	1 2 3 4 5
5.4	I experienced headache	1 2 3 4 5
5.5	I experienced blurred vision	1 2 3 4 5
5.6	I experienced dizziness (eyes closed)	1 2 3 4 5
5.7	I experienced vertigo	1 2 3 4 5

References:

1. Georgiou Y, Kyza EA. The development and validation of the ARI questionnaire: An instrument for measuring immersion in location-based augmented reality settings. International Journal of Human-Computer Studies. 2017;98:24-37.

2. Boletsis C. A User Experience Questionnaire for VR Locomotion: Formulation and Preliminary Evaluation. Lecture Notes in Computer Science: Springer International Publishing; 2020. p. 157-67.

3. Kim HK, Park J, Choi Y, Choe M. Virtual reality sickness questionnaire (VRSQ): Motion sickness measurement index in a virtual reality environment. Applied Ergonomics. 2018;69:66-73.

Investigator's Signature: ____________________ *Date:* ____________________
