# Supplementary material for: Assessment of Interrater Reliability and Accuracy of Cerebral Aneurysm Morphometry Using 3D Virtual Reality, 2D Digital Subtraction Angiography, and 3D Reconstruction: A Randomized Comparative Study
Source: Brain Sci. 2024 Sep 26;14(10):968. doi: 10.3390/brainsci14100968 (PMC11506597; doi:10.3390/brainsci14100968)
Supplement: Supplementary file 1 [file brainsci-14-00968-s001.zip › Supplemental Digital Content S2.pdf]

Supplemental Digital Content 1, Methods. Measurement case report form

Correlating size measurements of cerebral aneurysms based on Virtual Reality "Specto Medical" versus measurements based on standard 2D CTA/DSA – Case Report Form v2.0

**Patient_ID:**	☐☐☐	**Date:** (DD/MM/YYYY)	☐☐/☐☐/☐☐☐☐
**Investigator_ID:**	☐☐	**Modality Type:**	☐MRI ☐CT ☐VR

Instructions: *Diameters are to be measured to the nearest two decimal point. Time is to be measured from the point where measurement tool is first used*

**1. Aneurysm Measurements**		
1.1	**Anteroposterior diameter** of aneurysm (*maximum diameter measured in lateral view classified as an anteroposterior axis)* (AB, Figure 1): [(1)]	☐☐.☐☐mm
1.2	**Mediolateral diameter** of aneurysm (*Maximum mediolateral measurement determined on the anteroposterior view*): [(1)]	☐☐.☐☐mm
1.3	**Cephalocaudal diameter** of aneurysm *(maximum diameter measured in lateral view classified as a cephalocaudal axis)* (CD, Figure 1): [(1)]	☐☐.☐☐mm
1.4	**Smallest axial diameter / dome diameter** (*for aneurysms with complex shapes, the smallest axial diameter of the proximal dome is measured*) (b, Figure 2):[(2)]	☐☐.☐☐mm
1.5	**Maximum perpendicular height / depth** (*perpendicular to neck diameter plane*) (H1, Figure 3) [(3, 4)]:	☐☐.☐☐mm
1.6	**Maximum aneurysm height** (*maximum distance from the centroid of the aneurysm neck to any point on the aneurysm done, thus capturing the maximum deformation)* (H2, Figure 3) [(3), (5)]:	☐☐.☐☐mm
1.7	**Is a neck visible** (*only cross yes if a neck is definitely seen*)? [(1)]	☐Yes ☐No
1.8	**If a neck is visible, what is the diameter of the neck (N, Figure 3)?** [(3, 4)]	☐☐.☐☐mm
1.9	**Parent artery*** (*location is defined in terms of the junction of a secondary vessel arising from a parent vessel. When an aneurysm arises from such a junction, the name of the secondary vessel is used to label the aneurysm*) [(1),(6)]:	☐ MCA<br>☐ PCoA<br>☐ ACA<br>☐ ACoA<br>☐ BA bifurcation<br>☐ PICA<br>☐ ICA bifurcation<br>☐ ICA-OA<br>☐ ICA-ChA<br>☐ SCA<br>☐ VA
1.10	**What is the diameter of the parent vessel at the edge of the neck, perpendicular to flow** (D1, Figure 3)? [(3), (5)]	☐☐.☐☐mm
1.11	**What is the diameter of the parent vessel perpendicular to flow, measured at 1.5*D1 from D1** (D2, Figure 3)? [(3), (5)]	☐☐.☐☐mm

1.12	**Is the aneurysm a terminal aneurysm?** (*aneurysm arising off bifurcation of terminating feeding vessel)* [3]	☐Yes ☐No
1.13	**If the aneurysm is a terminal aneurysm, what are the mean vessel diameters of the feeding artery** (A, Figure 4) **and the branching arteries** (B, C, Figure 4) (*measured according to the method described in 1.10 and 1.11)* [5]	☐☐.☐☐mm (A-D1a)<br>☐☐.☐☐mm (A-D1b)<br>☐☐.☐☐mm (B-D2a)<br>☐☐.☐☐mm (B-D2b)<br><br>☐☐.☐☐mm (C-D3a)<br>☐☐.☐☐mm (C-D3b)
1.14	**How would you morphologically classify the aneurysm?** [1]	☐ A single sac with a smooth margin<br>☐ A single sac with an irregular corrugated margin<br>☐ A primary sac with a secondary daughter sac (*a separate protuberance arising from the main sac that is less than 25% of the total volume of the sac)* [1]<br>☐ A multilobed structure (*a protuberance arising directly from the primary neck of the aneurysm or arising from the main body and representing 25% or more of the apparent volume of the main sac)* [1]
1.15	**Orientation of the aneurysm:** [7]	☐ Inferolateral (IL)<br>☐ Lateral (L)<br>☐ Superolateral (SL)<br>☐ Inferomedial (IM)<br>☐ Medial (M)<br>☐ Superomedial (SM)<br>☐ Inferoposterior (IP)<br>☐ Posterior (P)<br>☐ Superoposterior (SP)
1.16	**Type of aneurysm:**	☐ Saccular (*aneurysm bulging on only one side of the artery wall*)<br>☐ Fusiform (*aneurysm bulging on both sides of the artery wall*)

1.17	**How would you treat the aneurysm?**	☐Craniotomy ☐Endovascular
**2. Time Measurement**		
2.1	**Time taken for measurements** (*time taken from moment DICOM data is loaded to final measurement being done*):	☐☐☐mins

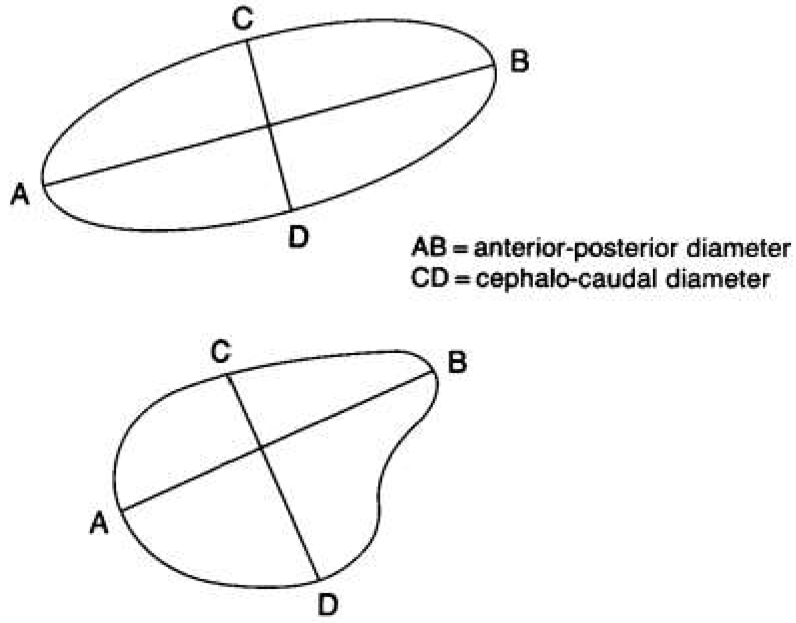

Figure 1: *Diameters of irregular or asymmetric aneurysms include maximum measurements in each plane*[(1)]

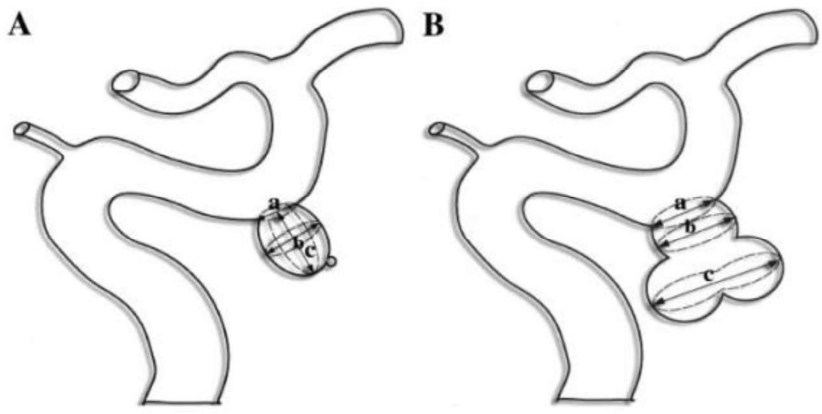

Figure 2: *Schematic drawings showing measurement of aneurysmal diameters in aneurysms with simple and complex shape.*[(2)]

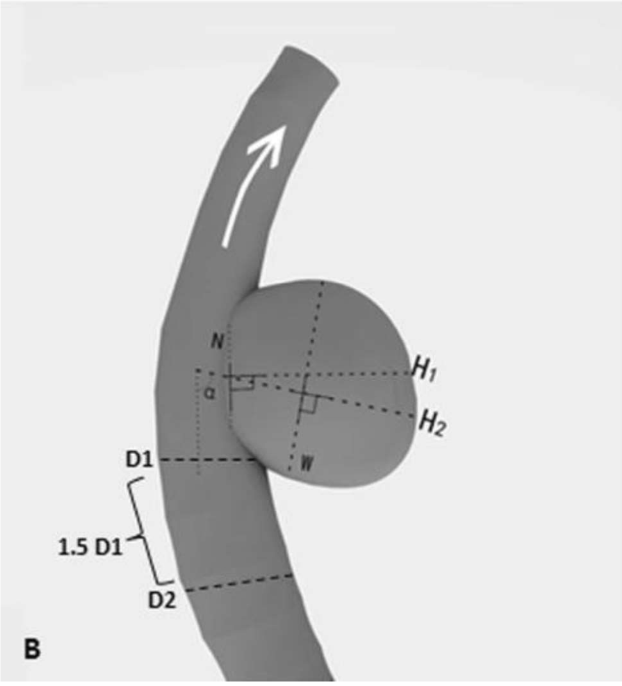

Figure 3: *Definition of the morphological parameters in side-wall aneurysm*[3]

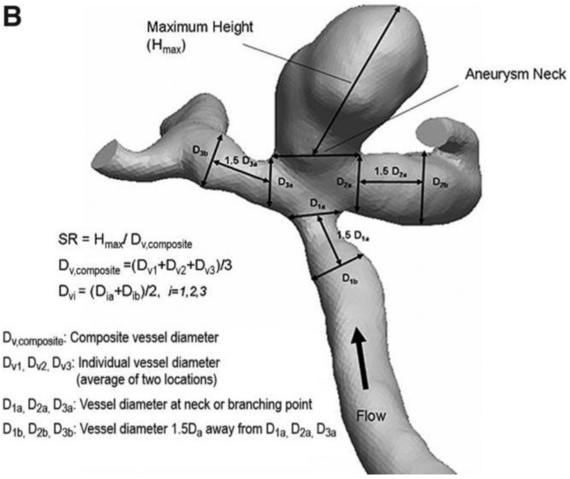

Figure 4: *Definition of size ratio (SR) for terminal aneurysms*[5]

Abbreviations:

**MCA = Middle cerebral artery; PCoA = Posterior communicating artery; ACA = Anterior communicating artery; ACoA = Anterior communicating artery; BA = Basilar artery; PICA = Posterior inferior cerebellar artery; ICA = Internal carotid artery; ICA-OA = Internal carotid artery – ophthalmic artery; ICA-ChA = Internal carotid artery – choroidal artery; SCA = Superior cerebellar artery; VA = Vertebral artery*

References:

1. Forbes G, Fox AJ, Huston J, 3rd, Wiebers DO, Torner J. Interobserver variability in angiographic measurement and morphologic characterization of intracranial aneurysms: a report from the International Study of Unruptured Intracranial Aneurysms. AJNR Am J Neuroradiol. 1996;17(8):1407-15.
2. Kiyosue H, Tanoue S, Okahara M, Hori Y, Nakamura T, Nagatomi H, et al. Anatomic features predictive of complete aneurysm occlusion can be determined with three-dimensional digital subtraction angiography. AJNR Am J Neuroradiol. 2002;23(7):1206-13.
3. Duan Z, Li Y, Guan S, Ma C, Han Y, Ren X, et al. Morphological parameters and anatomical locations associated with rupture status of small intracranial aneurysms. Scientific Reports. 2018;8(1).
4. Ujiie H, Tamano Y, Sasaki K, Hori T. Is the aspect ratio a reliable index for predicting the rupture of a saccular aneurysm? Neurosurgery. 2001;48(3):495-502; discussion -3.
5. Dhar S, Tremmel M, Mocco J, Kim M, Yamamoto J, Siddiqui AH, et al. Morphology parameters for intracranial aneurysm rupture risk assessment. Neurosurgery. 2008;63(2):185-96; discussion 96-7.
6. Korja M, Kivisaari R, Rezai Jahromi B, Lehto H. Size and location of ruptured intracranial aneurysms: consecutive series of 1993 hospital-admitted patients. Journal of Neurosurgery. 2017;127(4):748-53.
7. Sola T, Benítez E, Vivas E, Cuellar H, Nasis N, Guimaraens L. Clinical and Radiological Features of Posterior Communicating Artery Aneurysms. Interventional Neuroradiology. 2008;14(3):247-51.

Investigator's Signature: ____________________ *Date:* ____________________
